# Case Report: Phloroglucinol-Induced Kounis Syndrome

**DOI:** 10.3389/fcvm.2021.668318

**Published:** 2021-05-03

**Authors:** Hao-Yu Wu, Tian-Jiao Gao, Yi-Wei Cao, Peng-Hua You

**Affiliations:** ^1^Department of Cardiology, Shaanxi Provincial People's Hospital, Xi'an, China; ^2^Department of Gastroenterology, Xi'an Children's Hospital, Xi'an, China; ^3^Department of Electrocardiology, Shaanxi Provincial People's Hospital, Xi'an, China

**Keywords:** Kounis syndrome, allergy, phloroglucinol, chest pain, intravascular ultrasound

## Abstract

**Background:** Kounis syndrome is an allergy-related acute coronary syndrome that is induced by various pharmacological and environmental factors. Given that many clinicians are not aware of this condition, many cases may be underdiagnosed. We report a case of type II Kounis syndrome induced by phloroglucinol.

**Case Summary:** A 52-year-old man with pre-existing coronary artery stenosis presented with a 30-min history of chest pain and erythematous rash after intramuscular administration of phloroglucinol. An electrocardiogram demonstrated ST-segment elevation in leads II, III and aVF. Emergency coronary angiography revealed severe stenosis in the distal right coronary artery. Intravascular ultrasound showed plaque rupture and thrombosis, and the minimum lumen area was 3.0 mm^2^. A 3.5 × 38 mm stent was implanted in the distal right coronary artery. Troponin I levels were elevated. A diagnosis of type II Kounis syndrome induced by phloroglucinol was made, and the condition manifested as acute ST-segment elevation myocardial infarction.

**Conclusions:** Clinicians should be aware of Kounis syndrome as a possible diagnosis in a patient who presents with chest pain and allergic manifestations given that an increasing number of triggers are being reported.

## Introduction

Kounis syndrome is an acute coronary syndrome caused by mast cell and platelet activation induced by allergic reactions. At present, the diagnosis of Kounis syndrome is still based on the clinical manifestations and medical history of patients, and many cases may be missed or underdiagnosed. Although Kounis syndrome is rare, some triggers have been gradually reported, including drugs, medical conditions, and environmental exposures (1). Here, we report a case of Kounis syndrome induced by phloroglucinol.

## Case Presentation

A 52-year-old man presented to the emergency department with a 30-min history of chest pain. Prior to presentation, the patient had severe chest pain with transient loss of consciousness after intramuscular administration of 80 mg phloroglucinol to treat abdominal pain caused by right ureteral calculi. The patient's risk factors and past medical history included active tobacco use, diabetes mellitus for 6 years and treatment with insulin, and a known history of coronary artery disease. Two months earlier, the patient received stent implantation in the proximal right coronary artery (RCA), and the distal RCA showed 75% stenosis without percutaneous coronary intervention. He was taking aspirin, ticagrelor, and atorvastatin.

**Figure 1:**
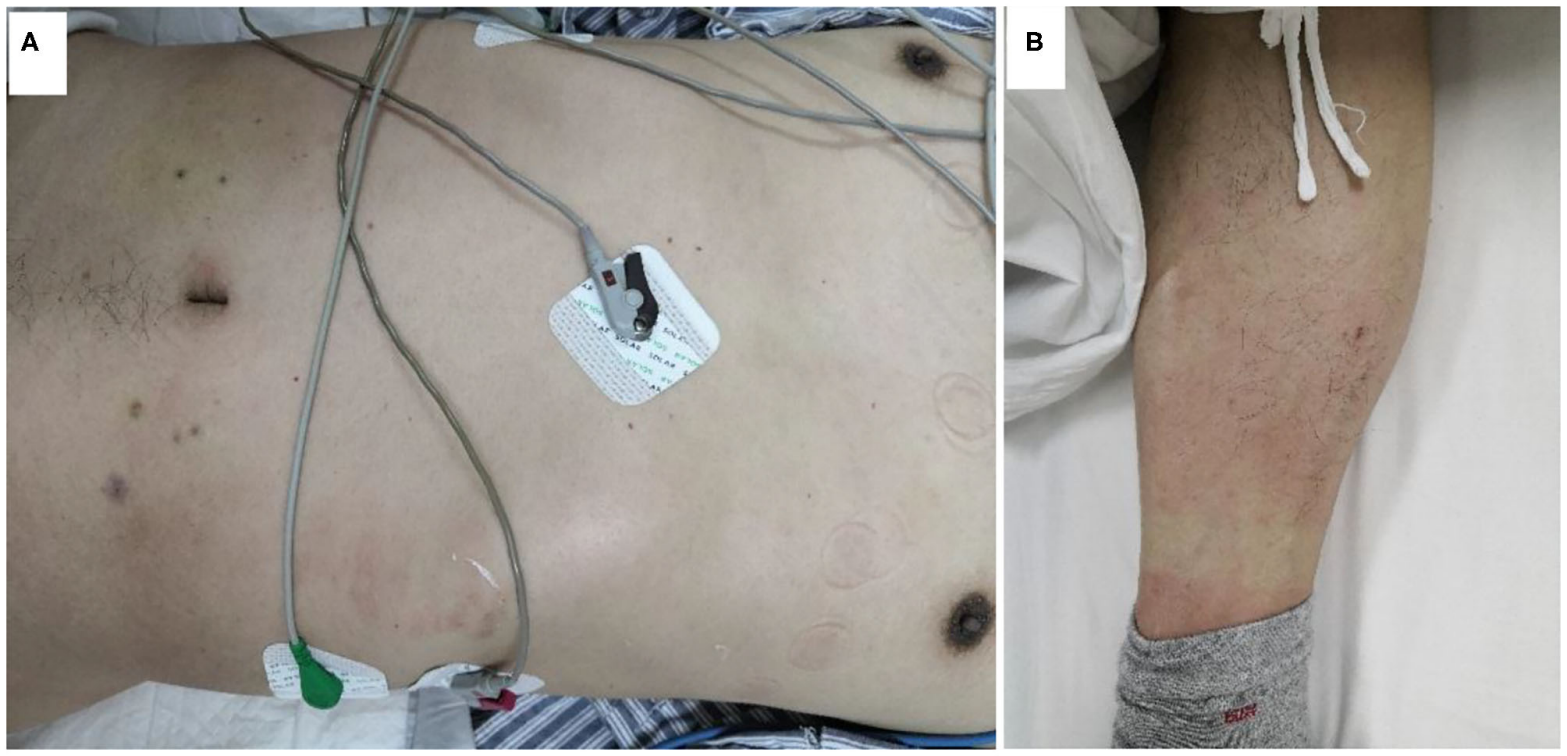
Erythematous rash over the chest, abdomen **(A)** and lower limb **(B)**.

**Figure 2:**
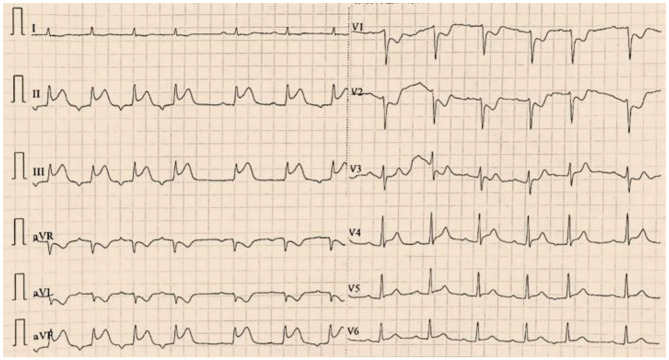
The first twelve-lead electrocardiogram indicated ST-segment elevation in leads II, III and aVF.

**Figure 3:**
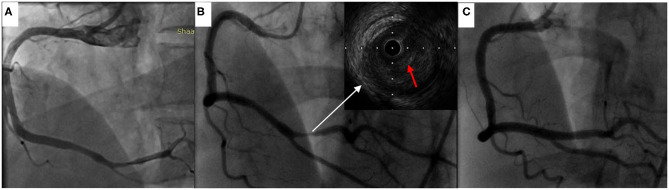
Coronary angiography and intravascular ultrasound findings. **(A)** The right coronary angiography results 2 months earlier. **(B)** Coronary angiography revealed severe stenosis in the distal right coronary artery, and intravascular ultrasound demonstrated plaque rupture and thrombosis (red arrow indicated thrombosis). **(C)** Coronary angiography after stent implantation showed good results.

**Figure 4:**
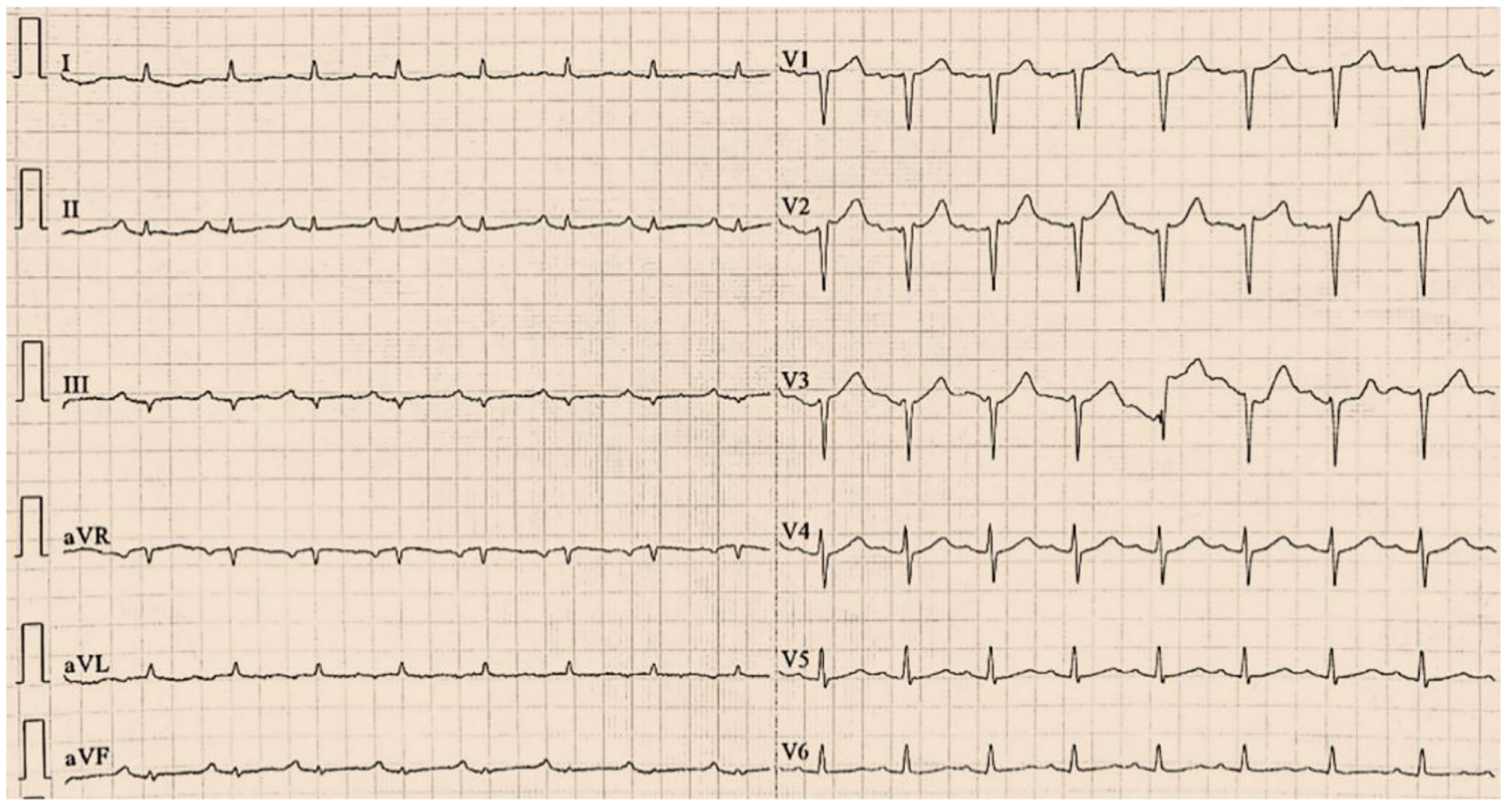
The postoperative electrocardiogram indicated ST-segment elevation in leads II, III and aVF disappeared.

Vital signs on arrival showed a blood pressure of 82/46 mmHg. Notably, erythematous rash was found throughout the chest, abdomen and limbs of the patient (Figure 1). The patient was first diagnosed with a systemic allergic reaction. We administered intravenous fluids and norepinephrine to maintain a blood pressure >90/60 mmHg. Dexamethasone (10 mg) and promethazine (25 mg) were administered to treat his allergic reaction. An initial twelve-lead electrocardiogram (ECG) immediately after admission showed ST-segment elevation in leads II, III and aVF (Figure 2). Acute inferior myocardial infarction due to RCA occlusion was also considered. Emergency coronary angiography was performed (the right coronary angiography results after stent implantation 2 months earlier was shown in Figure 3A), and the results revealed severe stenosis in the distal RCA (Figure 3B). Intravascular ultrasound showed plaque rupture and thrombosis (Figure 3B). The minimum lumen area at the stenosis was 3.0 mm^2^, and the plaque burden was 80.7%. Percutaneous coronary intervention was performed in the distal RCA with a 3.5 mm × 38 mm stent. Coronary angiography after stent implantation showed an acceptable angiographic result with a thrombolysis in myocardial infarction (TIMI) flow grade of 3 (Figure 3C). The procedure was successful, and the patient's symptoms were relieved completely. Postoperative ECG indicated ST-segment elevation in leads II, III and aVF disappeared (Figure 4). Laboratory evaluation revealed a troponin I level of 0.448 ng/ml (normal range <0.03), an eosinophil count of 0.51 × 10^9^/L (0.05–0.5), and an immunoglobulin E level of 405 IU/ml (0–100). Echocardiography revealed no obvious abnormality. The diagnosis of type II Kounis syndrome induced by phloroglucinol was made, and the condition manifested as acute ST-segment elevation myocardial infarction caused by allergic plaque rupture and thrombosis.

The patient received 100 mg/d aspirin, 180 mg/d ticagrelor, and 40 mg/d atorvastatin orally for myocardial infarction after percutaneous coronary intervention. The patient had an uneventful hospitalization and was discharged with a suggestion of avoiding phloroglucinol. During the 6-month follow-up, the patient did not experience chest pain. A detailed timeline from the onset of symptoms in the patient to his discharge is provided (Table 1).

**Table 1:** Timeline from the onset of symptoms in the patient to discharge is provided.

**Timeline**	**Symptom onset to discharge**
18 March 2020, 08:25	Developed abdominal pain
18 March 2020, 08:42	Intramuscular administration of phloroglucinol
18 March 2020, 08:51	Developed severe chest pain
18 March 2020, 09:22	Presented to the emergency department of our hospital
18 March 2020, 09:24	Initial twelve-lead ECG
18 March 2020, 09:55	Emergency coronary angiography
18 March 2020, 11:29	Postoperative ECG
24 March 2020, 15:10	Discharged

## Discussion

Phloroglucinol is a spasmolytic that is mainly used for gastrointestinal and urinary colic. It can inhibit the contraction of smooth muscle and relieve pain without anticholinergic side effects (2). In our case, the patient presented with chest pain and erythematous rash after intramuscular administration of phloroglucinol. Based on the clinical history, ECG, coronary angiography and intravascular ultrasound findings, and troponin I and immunoglobulin E levels, the diagnosis of type II Kounis syndrome induced by phloroglucinol was made. To our knowledge, this is the first report of type II Kounis syndrome induced by phloroglucinol.

Kounis syndrome is an acute coronary syndrome characterized by coronary artery spasm induced by allergic reactions, accompanied or not accompanied by erosion or rupture of atherosclerotic plaque. Patients who present with angina-equivalent symptoms (such as chest pain and dyspnea) and systemic allergic reaction (such as erythematous rash and wheezing) with or without a history of coronary heart disease should be suspected of Kounis syndrome (3). Clinical manifestations and medical history are the main diagnostic basis for Kounis syndrome. Blood biochemical tests (serum histamine, immunoglobulin E, eosinophils, tryptase and myocardial enzymes), echocardiography, ECG and coronary angiography results support the diagnosis of Kounis syndrome (4–6). The half-life of serum histamine is approximately 8 min. Therefore, a negative level of serum histamine does not exclude the diagnosis of Kounis syndrome. The application of immunoglobulin E levels in the diagnosis of Kounis syndrome is unclear, and a normal immunoglobulin E level cannot exclude the diagnosis of Kounis syndrome (7). Immunoglobulin E levels can also be elevated in patients with acute coronary syndrome (8). Clinicians should carefully review the patient's medical history, including drug use and allergic reactions, which is critical for the diagnosis of Kounis syndrome.

However, Kounis syndrome is not only a single organ disease but also a complex multisystemic and multiorgan arterial system involvement disorder affecting the coronary, mesenteric and cerebral arteries and is accompanied by allergy–hypersensitivity–anaphylaxis with involvement of the skin, respiratory and vascular systems. Kounis syndrome causes a wide range of clinical signs and symptoms in patients of any age through the release of inflammatory mediators during the interaction of allergic injury, postinflammatory cell activation and multidirectional stimuli (9).

According to the coronary angiography results, Kounis syndrome was divided into three variants. Type I, accounting for 72.6% of patients with Kounis syndrome, involves patients without obvious stenosis and coronary artery spasm caused by inflammatory factors. Type II, accounting for 22.3% of Kounis syndrome patients, involves patients with pre-existing coronary artery stenosis, and inflammatory factors cause coronary artery spasm together with atherosclerotic plaque rupture. Type III, accounting for 5.1% of Kounis syndrome patients, involves patients with coronary stent thrombosis (type III-a) or stent restenosis (type III-b) after stent implantation (10, 11). Our case was diagnosed with type II Kounis syndrome.

In 2015, Domínguez et al. first reported type III Kounis syndrome with intravascular ultrasound (12). Since then, the use of intracoronary imaging techniques (intravascular ultrasound or optical coherence tomography) in the diagnosis of Kounis syndrome are rarely reported. We believe that intracoronary imaging techniques should be an option in cases such as type II Kounis syndrome, where plaque rupture and thrombosis are expected to be found. Intracoronary imaging techniques can help us better understand Kounis syndrome and guide its treatment.

Presently, consensus guidelines on the treatment of Kounis syndrome are lacking. Many effective treatments are derived from case reports. H1 antihistamines, H2 antihistamines and corticosteroids are widely used to inhibit allergic reactions (13). Although epinephrine is a commonly used first-line drug to treat allergic reactions, it may aggravate coronary artery spasm and should be used with caution (14). Vasodilators, such as nitrates and calcium channel blockers, can relieve allergic vasospasms. The treatment of Kounis types II and III should also follow the latest guidelines for acute coronary syndrome (15, 16).

## Conclusions

Kounis syndrome is a rare and easily underdiagnosed disease that should be considered in some special clinical situations, such as angina-equivalent symptoms and systemic allergic reactions. Intracoronary imaging techniques can help us better understand Kounis syndrome.

## Data Availability Statement

The raw data supporting the conclusions of this article will be made available by the authors, without undue reservation.

## Ethics Statement

Written informed consent was obtained from the individual(s) for the publication of any potentially identifiable images or data included in this article.

## Author Contributions

All authors listed have made a substantial, direct and intellectual contribution to the work, and approved it for publication.

## Conflict of Interest

The authors declare that the research was conducted in the absence of any commercial or financial relationships that could be construed as a potential conflict of interest.
